# *N*′-(4-Bromo­benzyl­idene)quinoline-8-sulfonohydrazide

**DOI:** 10.1107/S1600536809008848

**Published:** 2009-03-19

**Authors:** Kely Navakoski de Oliveira, Ricardo José Nunes, Sabine Foro

**Affiliations:** a Departamento de Química–UFSC, 88040-900 Florianópolis, SC, Brazil; b Clemens Schöpf-Institut für Organische Chemie und Biochemie, Technische Universität Darmstadt, Petersenstrasse 22, D-64287 Darmstadt, Germany

## Abstract

In the title compound, C_16_H_12_BrN_3_O_2_S, the dihedral angle between the planes of the almost planar (r.m.s. deviation = 0.0263 Å) quinoline group and the bromo­phenyl group is 87.4 (1)°. The torsion angle of the central S—N—N—C bridge is 144.8 (2)°. The amino group has an intra­molecular contact to the quinoline N atom. The structure is stabilized by one N—H⋯O and two C—H⋯O inter­molecular hydrogen bonds.

## Related literature

For general background, see: Dueñas-Romero *et al.* (2006 ▶); da Silva *et al.* (2007 ▶). For related compounds, see: Oliveira & Nunes (2006 ▶); Silva *et al.* (2006 ▶).
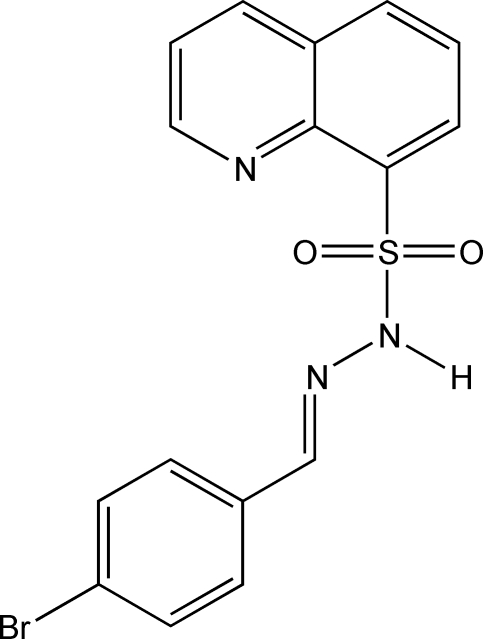


## Experimental

### 

#### Crystal data

C_16_H_12_BrN_3_O_2_S
*M* *_r_* = 390.26
Monoclinic, 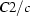

*a* = 32.149 (3) Å
*b* = 7.011 (1) Å
*c* = 16.589 (2) Å
β = 117.86 (1)°
*V* = 3305.7 (7) Å^3^
*Z* = 8
Cu *K*α radiation
μ = 4.68 mm^−1^
*T* = 299 K
0.65 × 0.28 × 0.10 mm

#### Data collection

Enraf–Nonius CAD-4 diffractometer
Absorption correction: ψ scan (North *et al.*, 1968 ▶) *T*_min_ = 0.147, *T*_max_ = 0.626
3889 measured reflections
2942 independent reflections
2677 reflections with *I* > 2σ(*I*)
*R*_int_ = 0.035
3 standard reflections frequency: 120 min intensity decay: 1.5%

#### Refinement

*R*[*F* ^2^ > 2σ(*F* ^2^)] = 0.052
*wR*(*F* ^2^) = 0.147
*S* = 1.05
2942 reflections
212 parameters
H atoms treated by a mixture of independent and constrained refinement
Δρ_max_ = 0.75 e Å^−3^
Δρ_min_ = −1.00 e Å^−3^

### 

Data collection: *CAD-4-PC* (Nonius, 1996 ▶); cell refinement: *CAD-4-PC*; data reduction: *REDU4* (Stoe & Cie, 1987 ▶); program(s) used to solve structure: *SHELXS97* (Sheldrick, 2008 ▶); program(s) used to refine structure: *SHELXL97* (Sheldrick, 2008 ▶); molecular graphics: *PLATON* (Spek, 2009 ▶); software used to prepare material for publication: *SHELXL97*.

## Supplementary Material

Crystal structure: contains datablocks I, global. DOI: 10.1107/S1600536809008848/im2087sup1.cif

Structure factors: contains datablocks I. DOI: 10.1107/S1600536809008848/im2087Isup2.hkl

Additional supplementary materials:  crystallographic information; 3D view; checkCIF report

## Figures and Tables

**Table 1:** Hydrogen-bond geometry (Å, °)

*D*—H⋯*A*	*D*—H	H⋯*A*	*D*⋯*A*	*D*—H⋯*A*
N1—H1N⋯O1^i^	0.89 (4)	2.18 (4)	2.959 (3)	146 (3)
N1—H1N⋯N3	0.89 (4)	2.36 (3)	2.887 (4)	118 (3)
C10—H10⋯O2^ii^	0.93	2.57	3.395 (4)	149
C12—H12⋯O2^ii^	0.93	2.50	3.361 (5)	154

Symmetry codes: (i) 
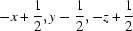
; (ii) 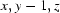.

**Fig. 1.**
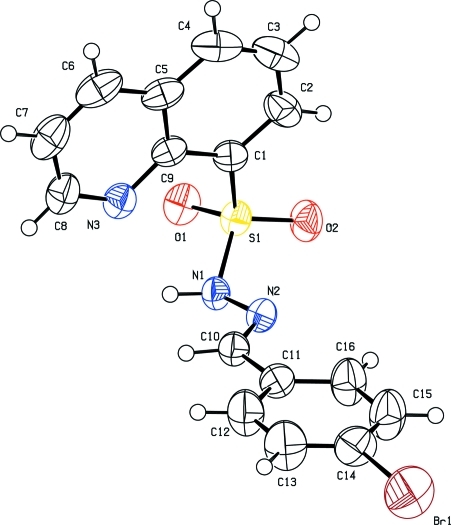
Molecular structure of (I), showing the atom labeling and displacement ellipsoids drawn at the 50% probability level.

**Fig. 2.**
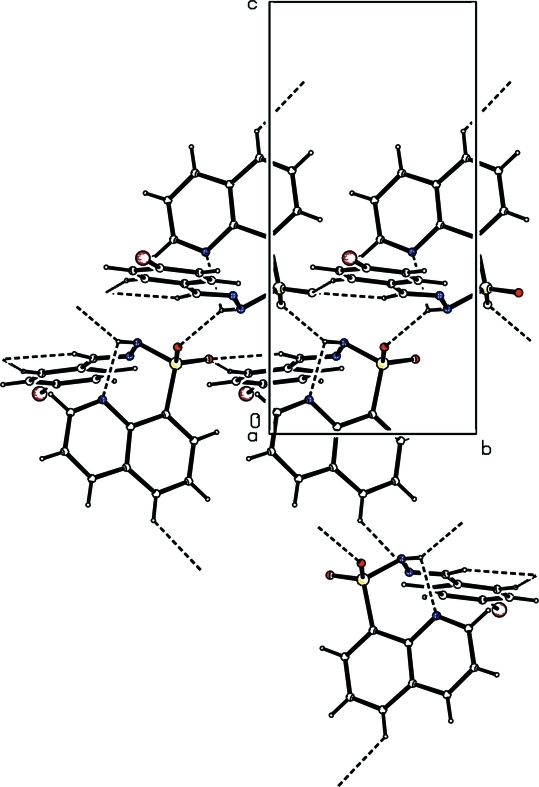
Molecular packing of (I) with hydrogen bonds shown as dashed lines.

## supplementary crystallographic information

### Comment

In the present work, the title compound has been synthesized to investigate its biological activity. Quinoline derivatives have been shown to be active against parasites. The quinine, for example, has been used as antimalarial drug years ago (Dueñas-Romero *et al.*, 2007). Recently, studies showed that quinoline sulfonic acid derivatives are active in assays with Leishmania spp (da Silva *et al.*, 2007). As part of our screening programs to investigate antiparasitic activity of quinoline derivatives, we report here a X-ray crystallographic study of the title compound (I). In the molecule of (I) (Fig. 1) the torsional angle of the central bridge S1—N1—N2—C10 is 144.8 (2)° and the plane of the quinoline ring system encloses a dihedral angle of 87.4 (1)° with the plane of the bromophenyl group. The NH group shows an intermolecular hydrogen bond towards the sulfonyl oxygen atom O1 [N—H···O = 2.18 (4) Å] and an intramolecular hydrogen bond to N3 [N—H···N = 2.36 (3) Å] leading to a bifurcated hydrogen bond. C10 and C12 have an intermolecular contact towards the sulfonyloxygen atom O2 [C—H···O = 2.57 (3) Å, C—H···O = 2.50 (3) Å, respectively] (Table 1).

### Experimental

The title compound (I) was synthesized by the reaction of *p*- bromobenzaldehyde (0.89 mmol, 160 mg) with quinoline-8-sulfonylhydrazide (0.89 mmol, 200 mg). The reaction was carried out in ethyl alcohol acidified with two drops of hydrochloric acid, as described for similar compounds (Oliveira *et al.*, 2006; Silva *et al.*, 2006). The mixture was stirred at room temperature for 4 h. After that, the solution was diluted with water and the resulting solid was filtered. The crystal used for data collection was obtained by dissolving 188 mg of (I) in 25 ml of ethyl alcohol and cooling down the solution to room temperature.

### Refinement

The CH atoms were positioned with idealized geometry using a riding model with C—H = 0.93 Å. The amino H atom was located in the difference map and was refined N—H = 0.89 (4) Å. The isotropic displacement parameters of all H atoms were set equal to 1.2*U*_eq_ (parent atom).

The residual electron-density features are located in the region of Br1. The highest peak and the deepest hole are 0.92 and 1.00 Å from Br1, respectively.

### Crystal data

**Table:**

C_16_H_12_BrN_3_O_2_S	*F*(000) = 1568
*M**_r_* = 390.26	*D*_x_ = 1.568 Mg m^−^^3^
Monoclinic, *C*2/*c*	Cu *K*α radiation, λ = 1.54180 Å
Hall symbol: -C 2yc	Cell parameters from 25 reflections
*a* = 32.149 (3) Å	θ = 3.1–20.6°
*b* = 7.011 (1) Å	µ = 4.68 mm^−^^1^
*c* = 16.589 (2) Å	*T* = 299 K
β = 117.86 (1)°	Long plate, colorless
*V* = 3305.7 (7) Å^3^	0.65 × 0.28 × 0.10 mm
*Z* = 8	

### Data collection

**Table:**

Enraf–Nonius CAD-4 diffractometer	2677 reflections with *I* > 2σ(*I*)
Radiation source: fine-focus sealed tube	*R*_int_ = 0.035
graphite	θ_max_ = 66.9°, θ_min_ = 3.1°
ω/2θ scans	*h* = −36→38
Absorption correction: ψ scan (North *et al.*, 1968)	*k* = −8→0
*T*_min_ = 0.147, *T*_max_ = 0.626	*l* = −19→4
3889 measured reflections	3 standard reflections every 120 min
2942 independent reflections	intensity decay: 1.5%

### Refinement

**Table:**

Refinement on *F*^2^	Secondary atom site location: difference Fourier map
Least-squares matrix: full	Hydrogen site location: inferred from neighbouring sites
*R*[*F*^2^ > 2σ(*F*^2^)] = 0.052	H atoms treated by a mixture of independent and constrained refinement
*wR*(*F*^2^) = 0.147	*w* = 1/[σ^2^(*F*_o_^2^) + (0.0805*P*)^2^ + 6.9219*P*] where *P* = (*F*_o_^2^ + 2*F*_c_^2^)/3
*S* = 1.05	(Δ/σ)_max_ = 0.007
2942 reflections	Δρ_max_ = 0.75 e Å^−^^3^
212 parameters	Δρ_min_ = −1.00 e Å^−^^3^
0 restraints	Extinction correction: *SHELXL97* (Sheldrick, 2008), Fc^*^=kFc[1+0.001xFc^2^λ^3^/sin(2θ)]^-1/4^
Primary atom site location: structure-invariant direct methods	Extinction coefficient: 0.00043 (7)

### Special details

**Table:**

Geometry. All e.s.d.'s (except the e.s.d. in the dihedral angle between two l.s. planes) are estimated using the full covariance matrix. The cell e.s.d.'s are taken into account individually in the estimation of e.s.d.'s in distances, angles and torsion angles; correlations between e.s.d.'s in cell parameters are only used when they are defined by crystal symmetry. An approximate (isotropic) treatment of cell e.s.d.'s is used for estimating e.s.d.'s involving l.s. planes.
Refinement. Refinement of *F*^2^ against ALL reflections. The weighted *R*-factor *wR* and goodness of fit *S* are based on *F*^2^, conventional *R*-factors *R* are based on *F*, with *F* set to zero for negative *F*^2^. The threshold expression of *F*^2^ > σ(*F*^2^) is used only for calculating *R*-factors(gt) *etc*. and is not relevant to the choice of reflections for refinement. *R*-factors based on *F*^2^ are statistically about twice as large as those based on *F*, and *R*- factors based on ALL data will be even larger.

### Fractional atomic coordinates and isotropic or equivalent isotropic displacement parameters (Å^2^)

**Table:**

	*x*	*y*	*z*	*U*_iso_*/*U*_eq_	
Br1	−0.070685 (17)	−0.11247 (10)	0.09298 (5)	0.0984 (3)	
S1	0.19475 (2)	0.54754 (10)	0.16299 (5)	0.0399 (2)	
O1	0.24468 (8)	0.5560 (3)	0.20074 (15)	0.0506 (6)	
O2	0.16963 (9)	0.7062 (3)	0.17320 (17)	0.0550 (6)	
N1	0.18486 (8)	0.3613 (4)	0.21089 (16)	0.0383 (5)	
H1N	0.2045 (13)	0.269 (6)	0.214 (2)	0.046*	
N2	0.13725 (8)	0.3233 (4)	0.18004 (16)	0.0401 (5)	
N3	0.20861 (9)	0.1911 (4)	0.07924 (17)	0.0454 (6)	
C1	0.17125 (10)	0.4981 (4)	0.04547 (19)	0.0406 (6)	
C2	0.14454 (13)	0.6347 (5)	−0.0160 (2)	0.0535 (8)	
H2	0.1358	0.7442	0.0039	0.064*	
C3	0.13046 (15)	0.6088 (6)	−0.1090 (3)	0.0670 (11)	
H3	0.1119	0.7007	−0.1508	0.080*	
C4	0.14362 (15)	0.4515 (6)	−0.1386 (2)	0.0646 (11)	
H4	0.1348	0.4384	−0.2003	0.077*	
C5	0.17042 (12)	0.3077 (5)	−0.0775 (2)	0.0501 (8)	
C6	0.18600 (15)	0.1403 (6)	−0.1035 (3)	0.0624 (10)	
H6	0.1789	0.1220	−0.1641	0.075*	
C7	0.21095 (14)	0.0087 (6)	−0.0407 (3)	0.0638 (10)	
H7	0.2216	−0.1004	−0.0573	0.077*	
C8	0.22084 (13)	0.0368 (5)	0.0498 (3)	0.0552 (8)	
H8	0.2370	−0.0587	0.0918	0.066*	
C9	0.18388 (10)	0.3272 (4)	0.01633 (19)	0.0405 (6)	
C10	0.12657 (11)	0.1475 (5)	0.1748 (2)	0.0424 (7)	
H10	0.1494	0.0554	0.1854	0.051*	
C11	0.07855 (12)	0.0890 (5)	0.1521 (2)	0.0467 (7)	
C12	0.06707 (15)	−0.1023 (6)	0.1405 (3)	0.0679 (11)	
H12	0.0894	−0.1918	0.1449	0.082*	
C13	0.02227 (16)	−0.1614 (7)	0.1221 (4)	0.0785 (12)	
H13	0.0145	−0.2903	0.1142	0.094*	
C14	−0.01013 (13)	−0.0307 (6)	0.1158 (3)	0.0639 (10)	
C15	0.00053 (14)	0.1602 (7)	0.1269 (3)	0.0758 (12)	
H15	−0.0220	0.2487	0.1224	0.091*	
C16	0.04473 (13)	0.2196 (6)	0.1446 (3)	0.0657 (10)	
H16	0.0519	0.3489	0.1516	0.079*	

### Atomic displacement parameters (Å^2^)

**Table:**

	*U*^11^	*U*^22^	*U*^33^	*U*^12^	*U*^13^	*U*^23^
Br1	0.0546 (3)	0.1028 (5)	0.1379 (6)	−0.0215 (3)	0.0451 (3)	0.0083 (3)
S1	0.0378 (4)	0.0351 (4)	0.0470 (4)	−0.0033 (3)	0.0202 (3)	−0.0071 (3)
O1	0.0393 (12)	0.0493 (13)	0.0586 (12)	−0.0131 (10)	0.0189 (10)	−0.0155 (10)
O2	0.0625 (14)	0.0372 (11)	0.0706 (14)	0.0061 (11)	0.0356 (12)	−0.0071 (10)
N1	0.0326 (12)	0.0390 (13)	0.0421 (12)	0.0041 (10)	0.0164 (10)	0.0014 (10)
N2	0.0346 (12)	0.0430 (14)	0.0435 (12)	0.0001 (11)	0.0189 (10)	−0.0027 (10)
N3	0.0453 (14)	0.0429 (14)	0.0519 (14)	−0.0017 (12)	0.0259 (12)	−0.0043 (11)
C1	0.0401 (15)	0.0382 (15)	0.0444 (15)	−0.0080 (12)	0.0206 (12)	−0.0009 (12)
C2	0.0531 (19)	0.0418 (17)	0.0601 (19)	−0.0021 (15)	0.0219 (16)	0.0094 (14)
C3	0.070 (3)	0.063 (2)	0.056 (2)	−0.0072 (19)	0.0195 (18)	0.0198 (18)
C4	0.071 (2)	0.077 (3)	0.0429 (17)	−0.024 (2)	0.0244 (17)	0.0044 (17)
C5	0.0496 (17)	0.061 (2)	0.0467 (16)	−0.0209 (16)	0.0282 (14)	−0.0089 (15)
C6	0.071 (2)	0.070 (2)	0.061 (2)	−0.027 (2)	0.0441 (19)	−0.0252 (19)
C7	0.065 (2)	0.060 (2)	0.082 (3)	−0.0125 (19)	0.047 (2)	−0.026 (2)
C8	0.0527 (19)	0.0480 (19)	0.074 (2)	−0.0014 (15)	0.0368 (17)	−0.0094 (16)
C9	0.0375 (14)	0.0438 (16)	0.0446 (15)	−0.0112 (13)	0.0227 (12)	−0.0029 (12)
C10	0.0392 (15)	0.0413 (16)	0.0469 (15)	0.0020 (13)	0.0203 (13)	0.0002 (12)
C11	0.0424 (16)	0.0472 (17)	0.0517 (16)	−0.0041 (14)	0.0231 (14)	0.0008 (13)
C12	0.055 (2)	0.048 (2)	0.104 (3)	−0.0022 (16)	0.039 (2)	0.0016 (19)
C13	0.062 (2)	0.053 (2)	0.121 (4)	−0.014 (2)	0.043 (2)	−0.002 (2)
C14	0.0454 (19)	0.070 (3)	0.075 (2)	−0.0129 (18)	0.0271 (17)	0.0046 (19)
C15	0.048 (2)	0.068 (3)	0.112 (3)	0.0004 (19)	0.038 (2)	−0.003 (2)
C16	0.0480 (19)	0.049 (2)	0.098 (3)	−0.0031 (17)	0.0332 (19)	−0.004 (2)

### Geometric parameters (Å, °)

**Table:**

Br1—C14	1.890 (4)	C5—C6	1.420 (5)
S1—O1	1.426 (2)	C6—C7	1.342 (6)
S1—O2	1.431 (2)	C6—H6	0.9300
S1—N1	1.635 (3)	C7—C8	1.395 (5)
S1—C1	1.765 (3)	C7—H7	0.9300
N1—N2	1.394 (3)	C8—H8	0.9300
N1—H1N	0.89 (4)	C10—C11	1.467 (4)
N2—C10	1.271 (4)	C10—H10	0.9300
N3—C8	1.320 (4)	C11—C12	1.380 (5)
N3—C9	1.362 (4)	C11—C16	1.382 (5)
C1—C2	1.369 (5)	C12—C13	1.389 (6)
C1—C9	1.421 (4)	C12—H12	0.9300
C2—C3	1.403 (5)	C13—C14	1.354 (6)
C2—H2	0.9300	C13—H13	0.9300
C3—C4	1.353 (6)	C14—C15	1.373 (6)
C3—H3	0.9300	C15—C16	1.374 (5)
C4—C5	1.403 (6)	C15—H15	0.9300
C4—H4	0.9300	C16—H16	0.9300
C5—C9	1.413 (4)		
			
O1—S1—O2	119.85 (14)	C6—C7—H7	120.3
O1—S1—N1	104.74 (14)	C8—C7—H7	120.3
O2—S1—N1	108.35 (14)	N3—C8—C7	123.7 (4)
O1—S1—C1	107.83 (14)	N3—C8—H8	118.1
O2—S1—C1	108.02 (15)	C7—C8—H8	118.1
N1—S1—C1	107.45 (13)	N3—C9—C5	123.0 (3)
N2—N1—S1	113.80 (19)	N3—C9—C1	119.2 (3)
N2—N1—H1N	121 (2)	C5—C9—C1	117.8 (3)
S1—N1—H1N	108 (2)	N2—C10—C11	120.5 (3)
C10—N2—N1	115.2 (3)	N2—C10—H10	119.8
C8—N3—C9	117.3 (3)	C11—C10—H10	119.8
C2—C1—C9	121.1 (3)	C12—C11—C16	118.9 (3)
C2—C1—S1	118.7 (3)	C12—C11—C10	119.2 (3)
C9—C1—S1	119.9 (2)	C16—C11—C10	121.8 (3)
C1—C2—C3	119.7 (4)	C11—C12—C13	120.2 (4)
C1—C2—H2	120.1	C11—C12—H12	119.9
C3—C2—H2	120.1	C13—C12—H12	119.9
C4—C3—C2	120.6 (4)	C14—C13—C12	119.8 (4)
C4—C3—H3	119.7	C14—C13—H13	120.1
C2—C3—H3	119.7	C12—C13—H13	120.1
C3—C4—C5	121.0 (3)	C13—C14—C15	121.0 (4)
C3—C4—H4	119.5	C13—C14—Br1	119.6 (3)
C5—C4—H4	119.5	C15—C14—Br1	119.5 (3)
C4—C5—C9	119.6 (3)	C14—C15—C16	119.5 (4)
C4—C5—C6	124.0 (3)	C14—C15—H15	120.2
C9—C5—C6	116.4 (3)	C16—C15—H15	120.2
C7—C6—C5	120.0 (3)	C15—C16—C11	120.7 (4)
C7—C6—H6	120.0	C15—C16—H16	119.7
C5—C6—H6	120.0	C11—C16—H16	119.7
C6—C7—C8	119.5 (3)		
			
O1—S1—N1—N2	−177.68 (19)	C8—N3—C9—C1	−178.1 (3)
O2—S1—N1—N2	53.3 (2)	C4—C5—C9—N3	177.9 (3)
C1—S1—N1—N2	−63.2 (2)	C6—C5—C9—N3	−2.9 (4)
S1—N1—N2—C10	144.8 (2)	C4—C5—C9—C1	−2.8 (4)
O1—S1—C1—C2	−113.8 (3)	C6—C5—C9—C1	176.4 (3)
O2—S1—C1—C2	17.1 (3)	C2—C1—C9—N3	−176.9 (3)
N1—S1—C1—C2	133.8 (3)	S1—C1—C9—N3	9.3 (4)
O1—S1—C1—C9	60.2 (3)	C2—C1—C9—C5	3.7 (4)
O2—S1—C1—C9	−168.9 (2)	S1—C1—C9—C5	−170.1 (2)
N1—S1—C1—C9	−52.2 (3)	N1—N2—C10—C11	173.8 (2)
C9—C1—C2—C3	−1.9 (5)	N2—C10—C11—C12	175.4 (3)
S1—C1—C2—C3	172.0 (3)	N2—C10—C11—C16	−6.5 (5)
C1—C2—C3—C4	−1.0 (6)	C16—C11—C12—C13	−0.4 (7)
C2—C3—C4—C5	2.0 (6)	C10—C11—C12—C13	177.7 (4)
C3—C4—C5—C9	0.0 (5)	C11—C12—C13—C14	−0.1 (7)
C3—C4—C5—C6	−179.1 (4)	C12—C13—C14—C15	0.3 (7)
C4—C5—C6—C7	−179.1 (3)	C12—C13—C14—Br1	−178.9 (4)
C9—C5—C6—C7	1.8 (5)	C13—C14—C15—C16	0.0 (7)
C5—C6—C7—C8	0.8 (6)	Br1—C14—C15—C16	179.2 (3)
C9—N3—C8—C7	1.8 (5)	C14—C15—C16—C11	−0.5 (7)
C6—C7—C8—N3	−2.8 (6)	C12—C11—C16—C15	0.8 (6)
C8—N3—C9—C5	1.2 (4)	C10—C11—C16—C15	−177.4 (4)

### Hydrogen-bond geometry (Å, °)

**Table:**

*D*—H···*A*	*D*—H	H···*A*	*D*···*A*	*D*—H···*A*
N1—H1N···O1^i^	0.89 (4)	2.18 (4)	2.959 (3)	146 (3)
N1—H1N···N3	0.89 (4)	2.36 (3)	2.887 (4)	118 (3)
C10—H10···O2^ii^	0.93	2.57	3.395 (4)	149
C12—H12···O2^ii^	0.93	2.50	3.361 (5)	154

Symmetry codes: (i) −*x*+1/2, *y*−1/2, −*z*+1/2; (ii) *x*, *y*−1, *z*.

## References

[bb1] Dueñas-Romero, A. M., Loiseau, P. M. & Saint-Pierre-Chazalet, M. (2006). *Biochim. Biophys. Acta*, **1768**, 246–252. 10.1016/j.bbamem.2006.07.003 16945323

[bb2] Nonius (1996). *CAD-4-PC* Nonius GmbH, Solingen, Germany.

[bb3] North, A. C. T., Phillips, D. C. & Mathews, F. S. (1968). *Acta Cryst.* A**24**, 351–359.

[bb4] Oliveira, K. N. de & Nunes, R. J. (2006). *Synth. Commun.* **36**, 3401–3409.

[bb5] Sheldrick, G. M. (2008). *Acta Cryst.* A**64**, 112–122. 10.1107/S0108767307043930 18156677

[bb6] Silva, L. L., de Oliveira, K. N. & Nunes, R. J. (2006). *Arkivoc*, **xiii**, 124–129.

[bb7] Silva, L. E. da, Joussef, A. C., Pacheco, L. K., Duarte, A. M. C., Steindel, M. & Rebelo, R. A. (2007). *Bioorg. Med. Chem.* **15**, 7553–7560. 10.1016/j.bmc.2007.09.007 17889546

[bb8] Spek, A. L. (2009). *Acta Cryst.* D**65**, 148–155. 10.1107/S090744490804362X PMC2631630 19171970

[bb9] Stoe & Cie (1987). *REDU4* Stoe & Cie GmbH, Darmstadt, Germany.

